# Tocotrienol in the Treatment of Topical Wounds: Recent Updates

**DOI:** 10.3390/pharmaceutics14112479

**Published:** 2022-11-16

**Authors:** Anroop B. Nair, Bapi Gorain, Manisha Pandey, Shery Jacob, Pottathil Shinu, Bandar Aldhubiab, Rashed M. Almuqbil, Heba S. Elsewedy, Mohamed A. Morsy

**Affiliations:** 1 Department of Pharmaceutical Sciences, College of Clinical Pharmacy, King Faisal University, Al-Ahsa 31982, Saudi Arabia; 2 Department of Pharmaceutical Sciences and Technology, Birla Institute of Technology, Mesra, Ranchi 835215, India; 3 Department of Pharmaceutical Sciences, Central University of Haryana, SSH 17, Jant, Mahendergarh 123031, India; 4 Department of Pharmaceutical Sciences, College of Pharmacy, Gulf Medical University, Ajman 4184, United Arab Emirates; 5 Department of Biomedical Sciences, College of Clinical Pharmacy, King Faisal University, Al-Ahsa 31982, Saudi Arabia; 6 Department of Pharmaceutical Sciences, College of Pharmacy, AlMaarefa University, Dariyah, Riyadh 13713, Saudi Arabia; 7 Department of Pharmacology, Faculty of Medicine, Minia University, El-Minia 61511, Egypt

**Keywords:** tocotrienols, topical wound, nanotechnology, challenges, growth factor, wound closure, angiogenesis

## Abstract

Healing wounds is an important attempt to keep the internal higher organs safe. Complications in topical wound healing may lead to the formation of scars, which can affect the patient’s quality of life. Although several approaches are ongoing in parallel in the exploration of natural compounds via advanced delivery, in this article, an attempt has been made to highlight tocotrienol. Tocotrienol is a natural form of vitamin E and has shown its potential in certain pharmacological activities better than tocopherol. Its antioxidant, anti-inflammatory, cell signal-mediating effects, angiogenic properties, management of scar, and promotion of wound environment with essential factors have shown potential in the management of topical wound healing. Therefore, this review has aimed to focus on recent advances in topical wound healing through the application of tocotrienols. Challenges in delivering tocotrienols to the topical wound due to its large molecular weight and higher logP have also been explored using nanotechnological-based carriers, which has made tocotrienol a potential tool to facilitate the closure of wounds. Exploration of tocotrienol has also been made in human volunteers for biopsy wounds; however, the results are yet to be reported. Overall, based on the current findings in the literature, it could be inferred that tocotrienol would be a viable alternative to the existing wound dressing components for the management of topical wounds.

## 1. Introduction

Emerging trends regarding disease-specific molecular alteration at the disease site have focused on agents for treatments. Topical wound treatment processes to combat diseases have shown gradual improvement over the years. The concept of Galen, the Greek physician, on wound healing environments by requiring a moist condition has been widely implemented during 120–201 AD [1,2,3]. Outcomes of further research on wound treatment brought another consequence of epithelialization in an occluded environment, where the time to heal the wound is shorter when the wound healing microenvironment along with the essential components was maintained, compared to the wounds left open and exposed to the environment [4,5]. Numerous research in recent years aims to bring novel agents in a new delivery tool to meet the requirements of ideal dressing [6]. Since the ancient period, exploring nature for herbal remedies against complex ailments brought us hope and tools to fight against several diseases [7,8]. The use of medicinal herbs as disease remedies has occupied a substantial market in the medicinal field [9]. Although there is an enormous exploration of these medicinal herbs, identification of the bioactives, pharmacological response, and toxicological aspects are not widely explored. Thereby, researchers are focusing on exploring standardized medicinal agents from plant sources for their consistent therapeutic response following appropriate mechanisms of action. In due course, the bioactives in the herbal sources are identified, extracted, and evaluated for their therapeutic efficacy, and authenticated. These herbal medicines in pure form have been brought to the bedside of patients with inflammatory disorders [10], dermatitis [11], psoriasis [12], diabetes [7], Alzheimer’s disease [13], cancer [14], etc. Concurrently, these herbs have shown enormous potential in the treatment of wound conditions, whether it is through cuts, scratches, breakdowns, or burns of the skin [9]. Since around 2% of the population in developing countries are suffering from different forms of wounds [15,16], it remains an important issue, particularly in high-risk patients [17].

One of the fat-soluble vitamins, vitamin E is referred to as a group of vitamins consisting of tocopherols and tocotrienols [18]. To combat the issues of healing wounds, without delaying or impairing the healing process, scarring and pain at the wound site, and creating stress to the patients, tocotrienol has shown huge potential in the improvement of patients’ quality of life [19]. The review is a summary of topical wounds and the role of tocotrienol in the healing of different types of wounds including the associated mechanism of healing and delivery aspects for prolonged efficacy in the wound environment. The connecting section of the article provides an overview of different types of topical wounds.

## 2. Topical Wounds

The primary function of the skin is to create a protective barrier to the environment to protect the inner organs of the body. Loss of integrity of a large portion of the skin, due to illness or due to injury, could lead to major incapacity or even death [20]. Based on the time frame of healing, wounds can be classified as acute or chronic. Repair of acute wounds requires a shorter period for healing. Such wound healing proceeds by itself. However, the degree of injury, including size and depth, could influence the healing process of acute wounds. Alternatively, the normal healing process is interrupted in chronic cases of wounds, where the healing process fails to follow a normal orderly, or timely manner [21].

On the other hand, based on the trigger factors, wounds can be classified as physical, thermal, or chemical wounds, which could result in interruptions or defects in the outermost layer of the skin or mucous membrane [22]. This mechanical category of wounds can be created by the application of blunt or sharp force on the skin. The blunt force can produce abrasions, where the outermost layer of the skin is abraded; bruises, or contusions, due to rupture of blood vessels of the skin or internal organs; and lacerations, where skin or muscle or mucous membrane or even internal organs can split off by the application of blunt force to a broad body area. On the other hand, sharp force injuries are cuts or incised injuries, which are superficial but to a larger surface, or puncture or penetrating or stab injuries which are due to penetration of sharp or pointed devices to the depth of the body [23]. Thermal or firearm injuries depend on various factors such as parts of the body, angle of firing, a distance of exposure, muzzle velocity, etc. [24]. Similarly, the degree and duration of exposure to corrosive chemicals, such as acids or alkalis, could produce injuries of a similar extent. Other categories of topical wounds can be created by exposure to electricity, lightning, or radioactive substances.

Whatever the type of wound that occurred on the skin, it is always challenging to repair and difficult to restore the skin in case of higher classes of wounds where the wound surface is prone to get an infection. The primary goal of treating such wounds would focus on rapid closure with the expression of aesthetical and functional scars on the surface of the wound. It had become possible through the understanding of the molecular pathways involved in healing wounds and by the application of novel treatments from natural herbal sources. The subsequent section of this article has included an introduction to tocotrienol, a natural lipophilic compound.

## 3. Tocotrienol: A Form of Vitamin E with a Superior Role

Although α-tocopherol is the well-known form of vitamin E, there are eight different varieties of this lipophilic agent that is available naturally. There are four different forms of tocopherols, α, β, γ, and δ, and their corresponding tocotrienols [25]. Structurally, these tocotrienols are unsaturated forms of vitamin E, whereas tocopherols are saturated forms [26]. According to the natural sources of vitamin E, tocopherols are ubiquitous, whereas sources of tocotrienols are specific to certain sources, such as rice bran, annatto bean, palm kernel, etc. Based on the metabolism of the synthetic pathways, the natural sources may contain varying compositions of different forms of tocotrienols and/or tocopherols [27]. For example, several studies have been performed on the beneficial role of tocotrienol-rich fraction (TRF), which can be obtained from palm oil. This fraction of palm oil contains different forms of tocotrienol (α, β, γ, and δ) as a major fraction (75%) while the rest 25% is the tocopherol (α-form). Alternatively, the percentage of δ-tocotrienol is much higher (90%) in annatto tocotrienol whereas around 10% is the γ-tocotrienol [28].

Alpha-tocopherol transfer protein (α-TTP), a liver cytosolic protein, helps transport vitamin E into the circulatory system. It plays an important role in maintaining the structural integrity of cells and reproductive function [29]. It is debatable whether the binding of α-TTP with other vitamin E isomers affects the bioavailability of the other isomers [18]. Consequently, the efficacy of tocotrienol (in mixture form) and α-tocopherol is controversial in treating different ailments [30].

**Figure 1:**
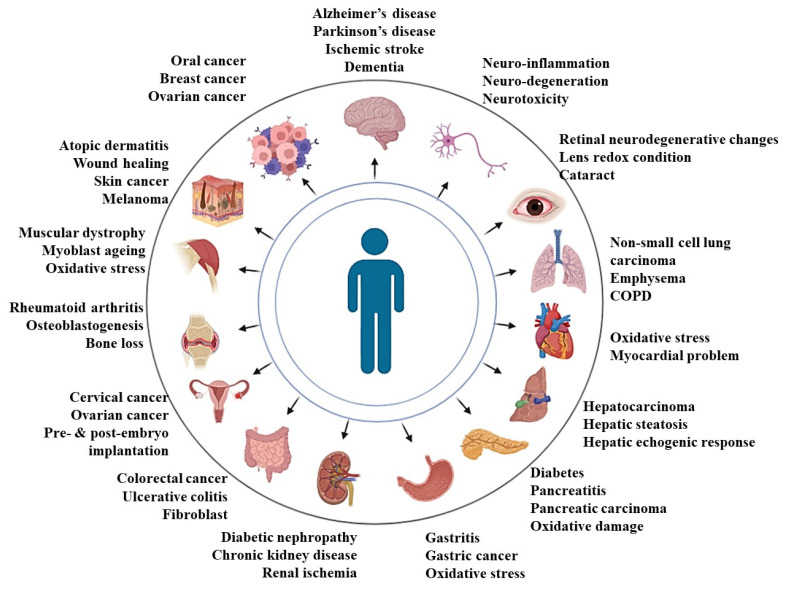
Presentation on the efficacy of tocotrienols in combating human diseases in preclinical studies.

Most of the research to date is focused on α-tocopherol, which is the ubiquitous form of vitamin E and the major form of vitamin E within human cells and tissues. However, gamma-tocopherol is the major form of vitamin E in the US diet [31]. Nevertheless, comparative studies available in the literature reveal better efficacy of tocotrienols compared to that of α-tocopherol in the management of several diseased conditions [32,33,34,35,36]. It has been postulated that the better antioxidant and related efficacy of tocotrienols might be due to the presence of unsaturated side chains in tocotrienol [32]. Overall, these tocotrienol forms of vitamin E, when supplemented, have been shown to attenuate a wide spectrum of diseases, which has been summarized in Figure 1 and Table 1.

### Types of Tocotrienols

**Figure 2:**
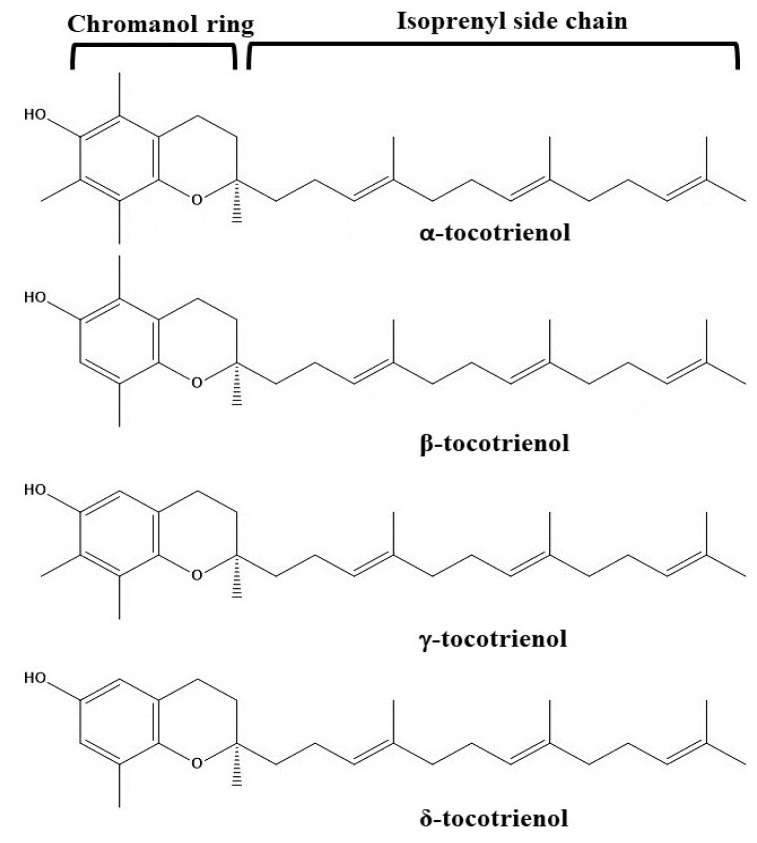
Chemical structures of different forms of tocotrienols.

The different sub-forms of tocotrienols structurally contain a chromanol ring with an unsaturated isoprenyl side chain at position 2 (Figure 2). The unsaturation positions of the side chain are situated between C3′-C4′, C7′-C8′, and C11′-C12′ [76,77]. Amongst the unsaturated positions, two of the positions C3′ and C7′ are possessing the *trans*-configuration [76]. Furthermore, different sub-forms (α-, β-, γ-, and δ-forms) are differing due to the position and number of methyl groups present on the chromanol ring. Different positions of the methyl group are presented in Figure 2. It is clear that the α-form of tocotrienol contains three methyl groups on the chromanol ring at the C5, C7, and C8 positions. On the other hand, β- and γ- forms contain two methyl groups at C5 and C8 positions and C7 and C8 positions, respectively. Lastly, the δ-tocotrienol contains only one methyl group at the C8 position.

Comparisons of the antioxidant potential of tocopherols and tocotrienols have been available in the literature, where it has been reported that the activity of the tocochromanols is largely depending on the assay environment. The alteration of activities is reported to change with a change in solvents [78]. In addition, it has also been established that the α-tocopherol subtype is the main subtype of vitamin E possessing anti-inflammatory and antioxidant effects; however, following comparison between γ-tocotrienol and α-tocopherol provided evidence of an advanced subtype of vitamin E, γ-tocotrienol, with stronger antioxidant and cell viability properties [65,79]. Detailed studies are yet to be published with the subtype of tocotrienol possessing superior properties.

The biological activities of tocotrienols are also attributed to the metabolites produced during the normal metabolism of the agent in the liver [80,81]. There is limited information available in the literature [82]. It has been postulated that tocotrienols are metabolized by oxidative degradation to the adjacent side chain. In this process, tocotrienols undergo ω-hydroxylation by the cytochrome P-450, followed by oxidization to ω-carboxylic acid. Thereafter, two carbon moieties of the structure are removed by the five cycles of β-oxidation [83]. Therefore, the final metabolites produced by the metabolism process are carboxyethyl hydroxychromans and their precursors, carboxymethyl butyl hydroxychromans [81]. For example, the δ-form of tocotrienol is metabolized to form various carboxychromanols (e.g., δ-tocotrienol-13-carboxychromanol). It is the major metabolite produced in the process of elimination through the feces of experimental animals [77,84]. This metabolite has shown the potential to inhibit two important enzymes, cyclooxygenases and 5-lipoxygenase. It has also shown its potential to impede tumor development in experimentally-induced colon cancer models [85]. Simultaneously, the role of tocotrienol has also been projected in the treatment of neural diseases [86], inflammatory disorders [87,88], osteoporosis [89], respiratory disorders [90], cancer [81,91], etc. Exploration of tocotrienols has also been made for topical application, dermatologically and cosmetologically [92,93]. Furthermore, a positive response of tocotrienols has also been reported while applied to surgical wound repair [94]. The upcoming section of the article consists of the mechanisms by which repair of the wound surface results from the action of tocotrienols.

## 4. Mechanisms of Tocotrienols in Wound Healing

**Figure 3:**
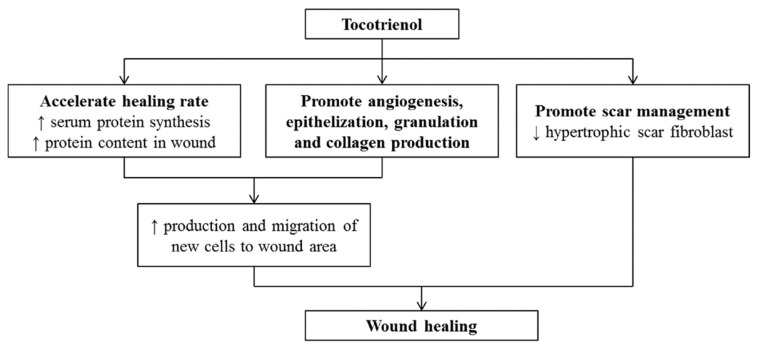
Effects of tocotrienols on wound healing (adapted from [106], published by MDPI, 2020).

Wounds are created by disrupting skin structure and functions [95]. Superficial wounds may lead to loss of epithelial cell integrity. The healing process of acute wounds occurs in an orderly manner as discussed earlier [96]. Disorders and disturbances in the healing of an acute wound prolong the healing of tissue, known as chronic wound healing. The prolonged healing process causes a financial and emotional burden on patients. Moreover, patients with diabetes, cancer, infection, and malnutrition are usually associated with abnormal healing processes [97,98]. It is evident from the literature that tocotrienols or their derivatives accelerate the wound-healing process when administered topically or orally [93,99,100]. According to the literature, wound contraction could be observed with accelerated healing rates [101]. This process was accomplished by enhanced migration and production of new cells in the damaged skin area with increased protein synthesis. Administration of tocotrienols increases serum protein synthesis and also enhances the protein content in the wound area to accelerate the healing process [102]. Tocotrienols are also known to contribute to the healing process by reducing oxidative stress markers through their endogenous antioxidant profile, encouraging epithelization, angiogenesis, granulation, and collagen production [103]. Furthermore, it also plays a potential role in scar management by hindering hypertrophic scar fibroblast [104]. However, inconsistent results for tocotrienol were observed in human trials for scar management due to poor penetration through the skin [105]. Overall, the mechanisms of healing topical wounds by the application of tocotrienols have been summarized in Figure 3.

Moreover, the literature suggests the potential of tocotrienols to expedite wound repair. However, further investigations or modifications are required to enhance the absorption of tocotrienols to prevent hypertrophic scarring in a clinical trial.

## 5. Application of Tocotrienols in the Treatment of Wounds: Recent Update

**Figure 4:**
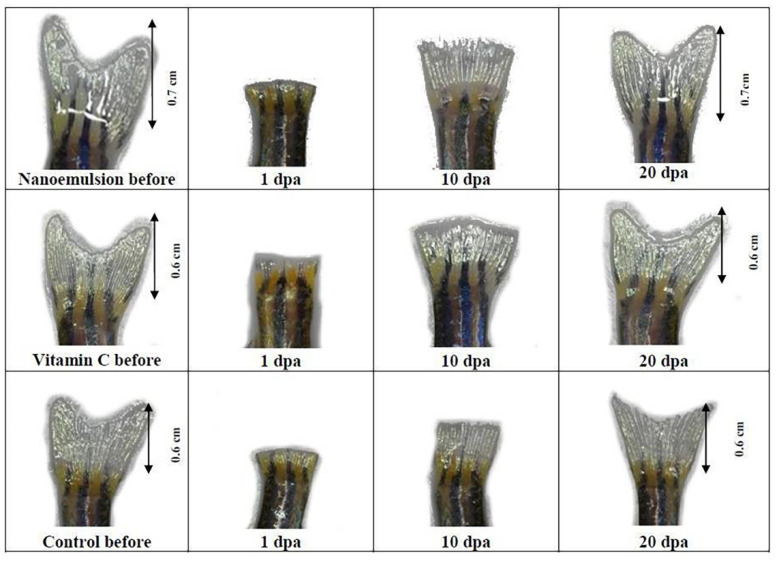
Adult zebrafish tail regeneration before and after treatments (adapted from [17], published by PLOS, 2022).

This section will emphasize recent research reported in the literature to investigate the wound-healing potential of tocotrienols. The antioxidant potential of tocotrienols is 60 times more potent than tocopherols [17]. It is well known for alleviating oxidative stress and inflammation in complications associated with diabetes. In the case of diabetic wounds, prolonged healing time and impairment in the healing process are the main setbacks in treatment. The main goal is a rapid closure of the diabetic wound; therefore, several strategies were explored for better treatment. With this aim, Yeo et al. explored the potential of tocotrienols-rich naringenin nanoemulgel for diabetic wound treatment. The optimized formulation showed uniformed dispersed globules (145.58 nm) with a narrow polydispersity index and negative zeta potential (−21.1 ± 3.32 mV). Moreover, nanoemulgel has good spreadability with a viscosity of 297,600 cP. A sustained release profile was observed with approx. 74% release of tocotrienols-rich naringenin from nanoemulgel. However, nanoemulsion showed higher release (89.17 ± 2.87%), which may attribute to the absence of polymer coating on disperse phase [107]. However, further studies are required to investigate the potential of nanoemulgel for diabetic wounds. Similarly, Chong et al. investigated the effectiveness of tocotrienol-based nanoemulsion on wound healing through zebrafish tail regeneration experiment and scratch assay. The MTT (3-[4,5-dimethylthiazol-2-yl]-2,5 diphenyl tetrazolium bromide) assay showed more than 100% cell viability when cells were treated with nanoemulsion (0.35–8.75 μg/mL) with a significantly faster rate of wound closure than in control groups. As shown in Figure 4, zebrafish tail growth was more rapid in the nanoemulsion (2.5 mg/mL) treated group than in the control group [17]. The 40%, 60%, and fully recovered regeneration was observed on the fifth day, the tenth day, and the twentieth day of nanoemulsion treatment, respectively. It concludes the potential of tocotrienol emulsified formulation for accelerated wound closure [17].

On the other hand, Hasan et al. investigated the safety of palm tocotrienol-rich fraction (TRF) nanoemulsion on the skin and ocular tissue. Nanoemulsion was prepared by high-pressure homogenizer with a droplet size of 137 nm and negative zeta potential (−24 mV) which indicates the stability of the formulation. The entrapment efficiency of TRF nanoemulsion was more than 80%. Additionally, irritation test on human corneal epithelium and reconstructed human epidermis indicates no irritancy to the eye or dermal tissue and are classified under Category 1 according to the United Nations Globally Harmonised System of Classification and Labelling of Chemicals [108].

Similarly, Xu et al. explored the beneficial effects of mono-epoxy-tocotrienol-α via in vitro and in vivo wound healing models. The scratch assay showed fast cell migration and wound closure on both high glucose and normal human fibroblast cells. Furthermore, endothelial tube formation was observed in human dermal microvascular endothelial cells. The results of microarray profiling analysis on HepG2 cells indicate 20 times enhancement in KIF26A gene expression along with 11 times reduction in lanosterol synthase expression. A significant increase in VEGFA and PDGFB (growth factors) was observed in expression analysis by qPCR. Additionally, a small but significant wound healing effect was observed on db/db mice (mouse model of phase 1 to 3 of type II diabetes and obesity) compared to placebo. This indicates the potential of mono-epoxy-tocotrienol-α on the diabetic wound by enhancement of gene expression, angiogenesis, cell growth, and motility [100].

Tocotrienol is well known for its antioxidant activity, which can suppress reactive oxygen species production, a leading cause of diabetic complications. Researchers have synthesized Deh-T3β, the modified form of tocotrienol, by sequential modification of geranylgeraniol and investigated the effect of this compound on diabetic complications, including wound healing. The results showed that Deh-T3β enhanced the insulin sensitivity of adipose tissue and positively impacted vital organs in diabetic mice with an improvement in mitochondrial function [103]. Likewise, Hoff et al. explored the potential of long-chain metabolites of vitamin E and garcinoic acid (GA), a δ-tocotrienol derivative, in wound healing. Different formulations loaded with active pharmaceutical ingredients were applied to the splinted mouse model wound. Results indicate accelerated wound healing and effective new tissue formation [109]. Another research group discovered the therapeutic potential of d-δ-tocotrienol rich fraction (d-δ-TRF) on diabetic wounds. Albino rats were treated with d-δ-TRF on full-thickness excisional skin wounds. The finding indicates the early regeneration of the dermis and epidermis with an increased serum protein level. It also helps control serum creatinine and blood glucose [110]. In another study, Elsy and Khan investigated the effect of vitamin E isoforms on stitched skin wounds in both healthy and diabetic rats. The rats were divided into normal control, diabetic control, and treated groups. Subcutaneous injection of alloxan was used to induce diabetes in rats and a horizontal skin incision was made then closed with a suture for wound creation. The treated groups were orally administered with d-α-tocopherol, d.-δ-TRF, and co-administration of both the vitamin E components. Results indicate better recovery of the wound with d.-δ-TRF treated groups via enhanced regeneration of epidermal and dermal components when compared to d-α-tocopherol and co-administered treated groups [111]. In contrast, another study reported the wound-healing activity of GA. Its activity was compared with those of long-chain metabolites of vitamin E (α-13′-COOH) in diabetic rats. The results indicate that both (GA and α-13′-COOH) significantly accelerated wound healing in a dose-dependent manner; however, GA did not show any effect on epidermal thickness compared to 13′-COOH. So, 13′-COOH was loaded in bacterial nanocellulose wound dressing to enhance the therapeutic effectiveness [109].

The majority of the wound treatment study of tocotrienol is on the diabetic model, but limited studies were conducted to consider the TRF in burn wounds. Therefore, Zaini et al. explored the antioxidant potential of TRF from palm oil in treating a partial thickness burn wound model. They formulated topical cream loaded with TRF, observed in the Sprague-Dawley rats’ burn model. Accelerated wound closure was observed with TRF treatment with stimulated granular tissue formation, rapid re-epithelialization, and regeneration. Moreover, TRF treatment also helps to decrease oedema, hyperaemia, and time of re-epithelization. However, further investigation is needed to know the mechanism of action of TRF on burn wound healing [112].

**Figure 5:**
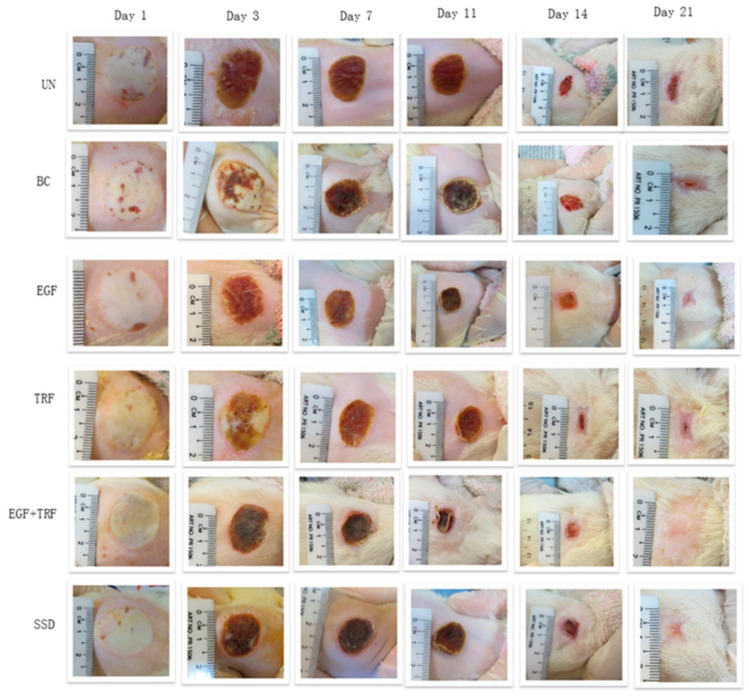
Wound closure of deep partial-thickness burn wounds over time in Sprague Dawley rats. (UN, without any treatment; BC, treated with base cream; Epidermal growth factor (EGF), treated with base cream containing c% EGF; Tocotrienol-rich fraction (TRF), treated with base cream containing 3% TRF; EGF + TRF, treated with base cream containing both c% EGF and 3% TRF; and SSD, treated with SSD cream) (adapted from [93], published by MDPI, 2020).

On the other hand, Guo et al. undertook the record of micro and macroscopic skin changes with burn wounds after being treated with TRF and epidermal growth factor (EGF). They found that the number of lymphocytes, neutrophils, and myofibroblasts was reduced post-burns after being treated with TRF + EGF cream; however, no effect was observed on adipose cell numbers. Moreover, post-burn oxidative stress was decreased with a reduction in nitrite production and lipid peroxidation. It is evident from Figure 5 that the rate of wound contraction is enhanced in a time-dependent manner; however, an insignificant difference in wound closure was observed on the 3rd day of treatment among all groups. EGF + TRF group treated group showed significant differences compared to untreated and treated with marketed formulation (SSD: Silverdin^®^ cream) on the 7th and 14th day post-burn. On the 21st day post-burn, 100% wound contraction was reached. This indicates that EGF + TRF has ameliorating effects on burn wound treatment [93].

In their subsequent study, they demonstrated the gene expression levels during the burn wound healing process. Similar to the previous study, Sprague Dawley rats were divided into positive control, negative control, and treated groups. The RNA samples were collected at different time points and subjected to quantitative real-time polymerase chain reaction to examine post-burn wound-healing genes. The downregulation of TNF-α, IL-6, and iNOS expression was reported in the complete healing process. In contrast, Collagen-1 expression was enhanced at the start of wound healing with no effect on the EGF receptor. It concludes that the combination of EGF + TRF accelerated the wound healing process by reducing inflammation and oxidative stress and enhancing tissue modeling of burn tissue [113].

Some researchers also explored the efficiency of tocotrienols for wound infection caused by Methicillin-resistant *Staphylococcus aureus* (MRSA). They extracted the tocotrienols from seeds of *Bixa orellana* and investigated the effectivity of daptomycin (DAP) with tocotrienols on mouse wound models with MRSA infection. The results demonstrated a lower bacterial load on the wound when treated with tocotrienol with DAP compared to DAP alone and the untreated group. This finding was supported by an increased level of the natural killer cell and markers of wound repair [114]. Other researchers also investigated the combination of α-tocopherol and γ-tocotrienol delivery via nanocarrier and microwave against dermatitis. The prepared nanoemulsion had droplet size in the nano-range (150 nm) with negative zeta potential. The skin was exposed to microwave before nanoemulsion application to enhance skin penetration. Pre-treatment with microwave-fluidized epidermis protein and lipid content leads to higher epidermal to the dermal distribution of nanoemulsion. Optimized fluidization was obtained with 3985 MHz of microwave [115]. Moreover, a clinical trial was conducted to evaluate the wound-healing effects of tocotrienol on healthy volunteers. A total of 101 participants participated to evaluate the efficacy of vitamin E on biopsy wounds. However, results were not disclosed yet [116]. The research findings along with the set research objectives have been summarized in Table 2.

All literature suggests the potential of tocotrienols in faster wound closure based on macro and microscopical observation and rapid epithelization. However, further studies are required to investigate the molecular mechanism of tocotrienols in wound healing and need more translational research to consider its use in humans.

## 6. Challenges of Tocotrienol Application

Vitamin E, either in the form of tocopherols or tocotrienols, is a lipid-soluble agent. Therefore, delivering this lipophilic agent to facilitate bioavailability is a great challenge. Concurrently, the hepatic first-pass metabolism of the absorbed fraction of the administered vitamin E further hinders the bioavailability of this agent. In addition to that, p-glycoprotein mediated efflux from the gastrointestinal epithelium contributes towards low oral bioavailability [115,117]. All these issues lead to poor oral bioavailability due to poor absorption and metabolism. Therefore, to target skin disorders, such as wounds or any inflammatory condition, it would be difficult to reach the site of action to fight against the diseased condition [118,119]. In such conditions, it is necessary to deliver the agents directly to the site of action, facilitating site-specific delivery, and avoiding the harsh condition of the gastrointestinal environment. This will further avoid alternative routes of administration of this lipophilic compound using invasive parenteral routes [107,120].

While applied topically, the outermost barrier of the skin, i.e., stratum corneum consisting of corneocytes attached by intercellular lipids, resists the penetration of the therapeutics similar to the unknown invaders [121]. Therefore, to facilitate the delivery of therapeutics across the barrier, active and passive delivery could be used. Active methods need external stimuli which facilitate the transportation of the therapeutics, such as ultrasonic and ultrasound techniques, iontophoresis and electroporation technologies, etc. [122,123,124]. Alternatively, the passive means of enhancing permeation involved the incorporation of permeation enhancers in the formulation or fabrication of nanocarriers for the therapeutics [6,121,125,126]. While combining these two approaches, a synergistic promotion could be achieved towards penetration of therapeutics across the skin barrier.

Several advanced delivery approaches have been made towards improved delivery of tocotrienols on topical application. Although the nanoparticular drug delivery approach has been most widely explored for almost all routes of drug administration, delivery of therapeutics on transdermal delivery faces a major drawback due to the presence of stratum corneum. Thus, this carrier has gained little success [127]. Alternatively, the vesicular delivery approach, liposome, has attained much focus due to the presence of lipidic bilayers on them which facilitate the transportation of lipid as well as aqueous soluble compounds using the same platform [128,129,130]. Several authors claim that the other vesicular approaches, such as ethosomes or transferosomes, are more efficient in permeating skin barriers to a deeper state due to the presence of ethanol or surfactant in the respective preparations [131].

Moreover, the higher molecular weight of the tocotrienol (γ-form) (410.6 g/mol) and the extreme hydrophobicity further resists the agent from easily permeating the outermost layer of skin. This problem can be overcome by fabricating a nanoemulsion of γ-tocotrienol. Application of nanoemulsion or via translating to nanoemulgel has widely been explored for lipophilic compounds [132,133]. Research results depicted superior permeation through the cellular-ester membrane [134]. To facilitate the evaluation of the permeability of the drug several approaches have been adopted, such as the incorporation of human skin or Strat-M^®^ membranes or cellulose ester membranes. Positive results towards penetration of tocotrienols using the advanced formulation approach suggest that the passage of the agents across the skin could be improved.

Furthermore, the use of polymers with special emphasis on using stimuli-responsive polymers has also been reported in the literature where the entrapped drug could be released in a sustained fashion to maintain the drug concentration at the topical wound [135,136]. Furthermore, in the case of in situ gel formulations, ease of application using spray technology forms a thin film over the application area and converts it into gel due to the conformational change in the polymeric structures [137]. However, the delivery of hydrophobic agents such as tocotrienols is a challenging task, but advancement of formulation-based research can counteract the associated issues easily.

## 7. Conclusions and Future Trend

Mostly pre-clinical evidence from the literature supports the therapeutic efficiency of tocotrienol in wound healing. Although limited clinical studies are reported on wound healing effects of tocotrienol, the wound healing efficiency of tocotrienol was evaluated on various types of wounds as a solo active pharmaceutical ingredient (API) or in combination with other API, entrapped in nanocarrier or the conventional dosage form in different preclinical assays. Cross-comparison of all evidence is complicated as researchers have used different combinations with tocotrienol and investigated their effect on different types of wounds such as open wounds, burn wounds, surgical wounds, etc. However, most of the research was conducted on the tocotrienol derivatives or in combination with another drug. The current evidence demonstrates that tocotrienol induced rapid regeneration of the dermis and epidermis, with enhanced gene expression, and angiogenesis. Moreover, studies on diabetic wound indicate that tocotrienol also contributes to the enhanced insulin sensitivity of adipose tissue and exert positive effects on vital organs in diabetic mice. The synergistic effect of tocotrienol on wound infection with antimicrobial agents was also noticed by some researchers. Most of the researchers attribute wound healing potential to the antioxidant and anti-inflammatory properties of tocotrienol. Still, more research may require establishing the molecular mechanism of wound healing with the safety profile of tocotrienol. There is a paucity of clinical trials on the wound-healing effect of tocotrienols in various types of wounds, although tocotrienol showed the potential to treat wounds in the current stage.

## Figures and Tables

**Table 1:** A summary of health effects reported with tocotrienols.

System Involved	Disease State	Possible Results	Source
Central nervous system	Alzheimer’s disease	Anti-inflammatory and prenylation-dependent AD synthesis helps in the prevention of Alzheimer’s disease	[37]
	Parkinson’s disease	Receptor-mediated cytoprotective effect helps in the early steps of Parkinson’s disease	[38]
	Ischemic stroke	Induction of Multidrug Resistance-Associated Protein 1 and arteriogenesis	[39,40]
	Dementia	Induction of platelet-derived growth factor-C in the hippocampus	[41]
	Neuro-inflammation	Preventing stress-mediated activation of the inflammatory cascade	[42]
	Neuro-degeneration	Reduction in inflammation, and prevention of reduction in dopaminergic neurons in the substantia nigra and striatum	[43]
	Neurotoxicity	Motor deficit prevention and neuroprotection	[44]
Sensory organ: Eye	Retinal neurodegenerative changes	Decreases retinal degenerative changes and an increase in VEGF expression	[45]
	Lens redox condition	≤0.1% decreases lens oxidative stress >0.1% increases lens oxidative stress	[46]
	Cataract	Delay onset of cataract by the reduction in lenticular oxidative and nitrosative stress (0.05–0.1%)	[46]
Sensory organ: Skin	Atopic dermatitis	Adjunct therapy for its antioxidant and anti-inflammatory efficacies	[47]
	Skin cancer	Chemonsensitization and anti-invasion effect against malignant melanoma Arrest cell cycle and facilitate apoptosis	[48,49]
Respiratory system	Non-small lung carcinoma	Induction of apoptosis and inhibition of cell growth	[50]
	Emphysema	Can decrease oxidative stress and airway inflammation	[51]
	Chronic obstructive pulmonary disease (COPD)	Targeting inflammatory pathway thereby modulate COPD progression	[51,52]
Cardiac system	Oxidative stress	Decrease heart oxidative stress and plasma homocysteine	[53]
	Myocardial ischemia	Mark ablation of oxidative stress, inflammation, and apoptosis	[54]
	Cardioprotective	Tocotrienol could improve dyslipidemia, inflammaging, and mitochondrial dysfunction	[55]
Digestive system	Gastritis	Controls gastric lesions better than alpha-tocopherol Decrease prostaglandin and decrease acidity	[56,57]
	Gastric cancer	It suppresses NF-κB–regulated markers of proliferation, angiogenesis, invasion, and metastasis	[58]
	Hepatic echogenic response	The hepatoprotective effect normalizes hepatic echogenic response	[59]
	Ulcerative colitis	Oral supplements could alleviate the severity of ulcerative colitis	[60]
	Fibroblast	Tocotrienol possesses an antifibrinogenic effect	[61]
	Colorectal cancer	Induce cytotoxicity and apoptosis Induces paraptosis-like cell death	[62,63]
	Pancreatitis	Can control pancreatitis by decreasing quantitative procedures of chronic pancreatic damage	[64]
	Pancreatic carcinoma	Facilitate apoptosis in pancreatic cancer cells by suppressing proliferative and cell signaling pathways	[65]
	Hepatocarcinoma	Antagonizes STAT3 activation pathway	[66]
Reproductive system	Embryo implantation	In vitro pre-implantation mice, embryos can be facilitated Also facilitate embryo development	[66,67]
	Ovarian cancer	It is a potent agent in chemotherapy-refractory ovarian cancer	[68]
	Cervical cancer	Induce apoptosis and possess a significant role in cancer cell physiology	[69]
Renal system	Renal ischemia	Application of tocotrienol can promote mitochondrial respiration, and tubular regeneration, decrease renal functions, and prevents ATP deficits, thereby increasing the survival of experimental animals after kidney injury	[70]
	Chronic kidney disease	Nephroprotective action facilitates the prevention of progressive chronic renal dysfunction	[71]
	Diabetic nephropathy	The renoprotective effect is reflected by the modulation of the release of profibrotic cytokines, ongoing chronic inflammation, oxidative stress, and apoptosis	[72]
Metabolic disorders and other cancers	Diabetes	Suppression of chronic inflammation, oxidative stress, and activation of peroxisome proliferator-activated receptors. Also increase insulin and glucose tolerance	[36]
	Obesity	Suppression of chronic inflammation, oxidative stress It is known to reduce body weight, fat mass, triglycerides, plasma concentrations of free fatty acid, and cholesterol	[36,73]
	Breast cancer	Provides synergy to the other chemotherapeutics Prevents the growth of breast cancer cells	[74]
	Oral cancer	Enhances the role of docetaxel through enhancement of chemosensitivity of this chemotherapeutic agent and facilitates apoptosis	[75]

**Table 2:** Glimpses of research conducted to investigate the potential of tocotrienols on the wound.

API/Formulation	Type of Wound Treated	Observations of the Research	Source
Tocotrienols/ nanoemulsion	Topical wound	Cell viability of more than 100% Faster zebrafish tail regeneration compared to the control group. Rapid wound closure	[17]
Tocotrienols-rich fraction/ nanoemulsion	Ocular and dermal tissue	Nanoemulsion droplet size 137 nm with a zeta potential of –24 Entrapment efficiency >80% Non-irritant to dermal and optical tissue	[108]
Mono-epoxy-tocotrienol-α	Diabetic wound	Enhancement of gene expression, angiogenesis, cell growth, and motility.	[100]
Dehydro-tocotrienol-β	Diabetic wound	Deh-T3β enhanced the insulin sensitivity of adipose tissue Exert positive effects on vital organs in diabetic mice Improvement in mitochondrial function Enhance wound healing	[103]
Long-chain metabolites of vitamin E, garcinoic acid	Diabetic wound	Accelerated wound healing and effective new tissue formation	[109]
d-δ-tocotrienol-rich fraction	Diabetic wound	Early regeneration of dermis and epidermis Increased level of serum protein. Controlling serum creatinine and glucose	[110]
d-δ-tocotrienol-rich fraction and α-tocopherol	Diabetic wound	δ-TRF treated groups via enhanced regeneration of epidermal and dermal components	[111]
Tocotrienols-rich fraction	Burn wound	Accelerate wound healing Decrease oedema, hyperemia, and time of re-epithelization	[112]
Tocotrienols and epidermal growth factor	Burn wound	Post-burn oxidative stress was decreased Reduction in nitrite production and lipid peroxidation Enhanced wound closure rate	[93]
Tocotrienols and epidermal growth factor	Burn wound	The downregulation of TNF-α, IL-6, and iNOS expression. Collagen-1 expression was enhanced with no effect on the EGF receptor	[113]
Daptomycin with Tocotrienols	Open wound	Lower bacterial load on the wound Increased level of the natural killer cell and markers of wound repair	[114]

## Data Availability

Not applicable.
